# SC-CoSF: Self-Correcting Collaborative and Co-Training for Image Fusion and Semantic Segmentation

**DOI:** 10.3390/s25123575

**Published:** 2025-06-06

**Authors:** Dongrui Yang, Lihong Qiao, Yucheng Shu

**Affiliations:** 1 Key Laboratory of Big Data Intelligent Computing, Chongqing University of Posts and Telecommunications, Chongqing 400065, China; 2022214597@stu.cqupt.edu.cn (D.Y.); shuyc@cqupt.edu.cn (Y.S.); 2 School of Artificial Intelligence, Chongqing University of Posts and Telecommunications, Chongqing 400065, China

**Keywords:** multimodal image fusion, semantic segmentation, self-correction, co-training

## Abstract

Multimodal image fusion and semantic segmentation play pivotal roles in autonomous driving and robotic systems, yet their inherent interdependence remains underexplored. To address this gap and overcome performance bottlenecks, we propose SC-CoSF, a novel coupled framework that jointly optimizes these tasks through synergistic learning. Our approach replaces traditional duplex encoders with a weight-sharing CNN encoder, implicitly aligning multimodal features while reducing parameter overhead. The core innovation lies in our Self-correction and Collaboration Fusion Module (Sc-CFM), which integrates (1) a Self-correction Long-Range Relationship Branch (Sc-LRB) to strengthen global semantic modeling, (2) a Self-correction Fine-Grained Branch (Sc-FGB) for enhanced visual detail retention through local feature aggregation, and (3) a Dual-branch Collaborative Recalibration (DCR) mechanism for cross-task feature refinement. This design preserves critical edge textures and color contrasts for segmentation while leveraging segmentation-derived spatial priors to guide fusion. We further introduce the Interactive Context Recovery Mamba Decoder (ICRM) to restore lost long-range dependencies during the upsampling process; meanwhile, we propose the Region Adaptive Weighted Reconstruction Decoder (ReAW), which is mainly used to reduce feature redundancy in image fusion tasks. End-to-end joint training enables gradient propagation across all task branches via shared parameters, exploiting inter-task consistency for superior performance. Experiments demonstrate significant improvements over independently optimized baselines in both fusion quality and segmentation accuracy.

## 1. Introduction

In the field of autonomous driving, precise and robust scene analysis is crucial. However, in complex environments (such as adverse weather or nighttime driving), relying solely on visible light sensors may make it difficult to accurately identify targets [1,2,3]. In contrast, infrared sensors are not affected by these factors and can provide clear target information in low light and harsh weather conditions; however, their spatial resolution is lower. Therefore, infrared and visible modality fusion (IVMF) has become an effective approach to enhance scene understanding and target detection performance [4,5].

From a practical application perspective, IVMF aims to generate high-quality, visually perceptive fused images, along with high-precision semantic segmentation results for scene analysis tasks. In recent years, advances in deep learning have significantly driven the development of the IVMF field [6,7,8,9]. For instance, CDDFuse [10] cleverly combines the global feature extraction capability of Transformers with the local feature-capturing advantages of CNNs, achieving efficient fusion of multimodal images. In the field of semantic segmentation, SegFormer [11] outputs multi-scale features by adopting a hierarchical structure Transformer encoder, without the need for positional encoding, thereby avoiding performance degradation when the test resolution differs from the training resolution. Although the above methods have achieved encouraging results in terms of visual effects and segmentation performance, there are still several challenges.

First, in semantic segmentation, although the Transformer-based global context modeling capability has shown remarkable results, its high computational complexity makes it difficult to fully leverage its advantages on low-performance devices, which can lead to insufficient feature aggregation. Additionally, during the upsampling process, some global context modeling ability may be lost, weakening the model’s ability to make semantic decisions for continuous regions. At the same time, the introduction of the Mamba architecture has brought innovation to the field [12], offering hope for solving the existing problems. Mamba excels at efficiently capturing global context information and is renowned for its computational efficiency based on the State Space Model (SSM) [13]. Vision Mamba [14] and VMamba [15] extended this architecture to computer vision tasks, further enhancing performance by strengthening the unidirectional scanning mechanism.

**Figure 1:**
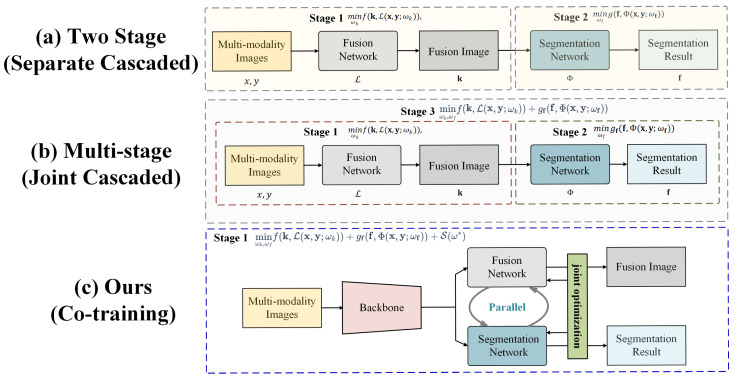
Compared to previous fusion-segmentation frameworks, our method redefines the framework by integrating image fusion and semantic segmentation subnetworks. By introducing a joint training mechanism that optimizes both tasks simultaneously, our model is optimized using synchronous single-stage joint optimization. This parallel training structure effectively enhances the performance of both tasks during joint training, reduces training difficulty, and eliminates the performance bottleneck between tasks.

Secondly, in image fusion, the limited receptive field of CNNs, due to finite kernel sizes, causes local artifacts and hampers the preservation of fine-grained details, reducing visual quality. Additionally, the weight-sharing mechanism across regions makes CNNs prone to learning redundant feature representations, leading to feature redundancy. Finally, most existing methods treat image fusion and semantic segmentation as independent problems, overlooking the inherent correlation between the two. Although some studies [16,17] validate the effectiveness of fused images by using the results of image fusion as input for semantic segmentation or object detection, as shown in Figure 1, this cascading approach lacks effective strategies to fully utilize the inherent consistency between the two, failing to maximize their potential. At this point, multi-task learning (MTL) [18], which has been widely applied in the field of machine learning, inspired us. This approach aims to construct a unified model capable of simultaneously addressing multiple related tasks, with the goal of leveraging the underlying commonalities among tasks to enhance overall performance and reduce inefficiencies and redundancies during training [19,20]. In particular, MTL has demonstrated significant potential and promising prospects in dense prediction tasks such as semantic segmentation [21,22,23].

To address the aforementioned challenges and inspired by recent advances in state-of-the-art technologies, in this paper, we propose SC-CoSF, a novel coupled framework for image fusion and semantic segmentation. This model leverages the similarity between the two tasks, treating them as a joint task to promote mutual benefits rather than handling them independently. On this basis, we design the Self-correction and Collaboration Fusion Module (Sc-CFM), which comprises a Self-correction Long-Range Relationship Branch (Sc-LRB), a Self-correction Fine-Grained Branch (Sc-FGB), and a Dual-branch Collaborative Recalibration (DCR) mechanism. The Sc-LRB employs Mamba-based global attention and cross-attention to effectively capture cross-region dependencies and enhance semantic modeling, thereby improving edge sharpness and structural coherence; the Sc-FGB integrates multi-scale convolution operations to extract fine texture details and enrich visual information; and the DCR mechanism fuses the outputs of these two branches to dynamically reshape visual features and semantic information flows, ensuring that the fused results exhibit both high contrast and rich spatial semantics. Finally, we introduce two task-specific decoders. The Interactive Context Restoration Mamba Decoder (ICRM) for semantic segmentation recovers remote contextual information lost during upsampling. The Region-Adaptive Weighted Reconstruction Decoder (ReAW) for image fusion adaptively suppresses redundant features to enhance the visual quality of the fused image. By jointly optimizing the above tasks, the encoded features propagate gradients across all task branches through shared parameters, fully utilizing the inherent consistency between tasks and achieving better performance than independent optimization.

To summarize, our main contributions are as follows:

- We design a Self-correction and Collaboration Fusion Module (Sc-CFM), which integrates a Self-correction Long-Range Relationship Branch (Sc-LRB) to enhance semantic modeling capability and a Self-correction Fine-Grained Branch (Sc-FGB) to comprehensively capture multi-scale visual information, and employs a Dual-branch Collaborative Recalibration (DCR) mechanism to simultaneously recalibrate semantic features and visual representations. The Sc-CFM is specifically engineered to autonomously optimize the embedded semantic and visual features within latent representations, while simultaneously facilitating synergistic fusion between enhanced semantic guidance and raw visual signals. This dual-phase refinement process not only suppresses feature-level noise but also reinforces cross-modal alignment, ultimately generating discriminative feature representations that substantially improve downstream task performance.
- We propose an Interactive Context Restoration Mamba Decoder and a Region-adaptive Weighted Reconstruction Decoder, aiming to recover remote information lost during the upsampling process and reduce feature redundancy in the fused image.
- By establishing a coupling module that integrates image fusion with semantic segmentation in a complementary manner, our approach effectively harnesses the strengths of both tasks, thereby delivering a performance that maximizes each modality’s advantages.

## 2. Related Work

### 2.1. Multi-Modality Fusion and Segmentation

Recent deep learning-based multimodal image fusion methods have made significant progress. Early attempts [24,25,26] focused on adjusting network structures or loss functions to achieve fusion results, but often overlooked downstream task performance. Some fused images with good metrics may not be suitable for practical applications. Zhang et al. [27] introduced the CMX framework to improve semantic segmentation by integrating RGB and other modalities using cross-modal calibration and fusion modules. Tang et al. [16] proposed SeAFusion, which incorporates semantic segmentation feedback to balance low-level visual details and high-level semantic information, generating fused images that retain both thermal and texture details. Semantic segmentation and image fusion are jointly trained in an integrated manner. Liu et al. [17] achieved collaborative fusion of textures and semantics from infrared and visible images, leveraging mutual promotion to improve detail restoration and semantic representation. Jiang et al. [28] introduced domain adaptation branches to capture semantic and geometric information, bridging the gap between modalities and improving fused image quality for downstream tasks. However, these fusion-segmentation methods often rely on cascaded architectures, resulting in complex training and lacking an end-to-end design. As a result, they struggle to produce reliable fused images and accurate segmentation outputs with a single unified network.

### 2.2. State Space Models

State Space Models (SSMs) [29], inspired by linear time-invariant systems, have emerged as highly efficient sequence-to-sequence architectures. In particular, the Structured State-Space Sequence model (S4) [13] has been recognized as a groundbreaking framework for capturing long-range dependencies. Building on this success, the incorporation of selective mechanisms into S4, as demonstrated by Mamba [12], has enabled it to outperform Transformers and other state-of-the-art architectures. Capitalizing on these impressive results, researchers have recently extended SSMs to computer vision applications. For example, Vision Mamba [14] combines SSMs with bidirectional scanning to establish robust inter-patch relationships, and VMamba [15] further enhances this approach by incorporating four-directional scanning, thereby more comprehensively capturing the interrelations among image patches. Inspired by the above methods, we incorporate State Space Models (SSMs) into our multimodal task and design a Mamba-based fusion strategy. We also propose a dual-branch Mamba decoder for information selection. This approach balances low computational complexity and high accuracy by integrating the SSM mechanism into the fusion process.

### 2.3. Multi-Task Learning

Multi-task learning (MTL) seeks to enhance the performance of several related tasks by sharing and utilizing relevant information across them. Various approaches have been proposed to achieve effective multi-task learning by modifying the model architecture. For instance, Felix et al. [30] designed the architecture by using a cross-task sharing approach at the lower layers of the model. Ishan Misra et al. [31] proposed a novel module that enhances multi-task learning performance by learning a linear combination of activation maps from different tasks, allowing the network to find an optimal balance between shared and task-specific representations. Meanwhile, many emerging techniques for multi-task learning have also been proposed. For example, Zhang et al. [32] achieve mutually beneficial closed-loop learning by adaptively enhancing complementary information between tasks through the use of a task attention module (TAM). Other methods approach multi-task learning from an optimization perspective, combining task objectives into weighted sums [33,34], gradient normalization [35,36], and dynamic adjustment of task importance [17]. These methods further enhance the collaboration between tasks. Inspired by the above methods, we have incorporated the concept of multi-task learning into our framework design. By leveraging low-level feature sharing and a weighted loss function, we effectively utilize the inherent consistency between different tasks, mitigating the performance trade-off problem in multi-task learning.

## 3. Methodology

### 3.1. Problem Formulation

For tasks such as multi-modal image fusion or segmentation, a widely adopted method is to design a neural network that fully utilizes its capacity to determine an optimal set of parameters The optimization model is formulated as follows:

(1) $$\min_{\omega_{k}}f\left(k,L\left(x,y;\omega_{k}\right)\right),$$

where k represents the output of the task-specific network L with learnable parameters $\omega_{k}$. f(·) is a constraint term used to optimize the network. Here, we suppose a pair of an RGB image $x\in R^{H\times W\times C_{x}}$ and an infrared image $y\in R^{H\times W\times C_{y}}$, respectively.

The previous methods mostly designed image fusion and semantic segmentation frameworks in a cascading manner, making it difficult to find a balance between different tasks. Therefore, here, we design a coupling learning framework to integrate the objectives of different tasks, which can be written in the following form:

(2) $$\min_{\omega_{f},\omega_{s}}g_{f}\left(f,\Phi (x,y;\omega_{f})\right)+h_{s}\left(s,\Psi (x,y;\omega_{s})\right)+S\left(\omega^{★}\right)$$

where $\omega^{★}=\left\{\omega_{f},\omega_{s}\right\}$ denotes the parameters of the fusion network Φ and segmentation network Ψ, respectively, with some parameters shared between them. f and s denote the fused image and segmentation results, which are produced by the fusion network Φ and Ψ. S(·) is a constraint term for joint optimization, implemented via a bespoke distance-based loss.

### 3.2. Overvall Architecture

**Figure 2:**
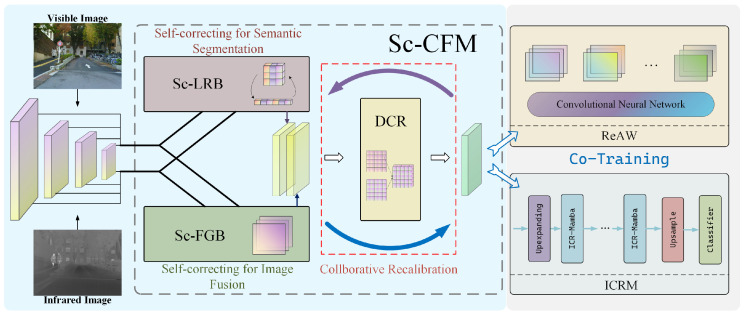
The overall framework of our proposed SC-CoSF.

The proposed SC-CoSF is designed using a parallel strategy, consisting of image fusion and semantic segmentation subnetworks. The overall structure details are shown in Figure 2. Sc-CFM effectively extracts and couples semantic and visual information from multimodal data, while the segmentation network (ICRM) and fusion network (ReAW) extract key information from the coupled features, fully capturing detailed information and extracting semantic features.

#### 3.2.1. Sc-CFM

**Figure 3:**
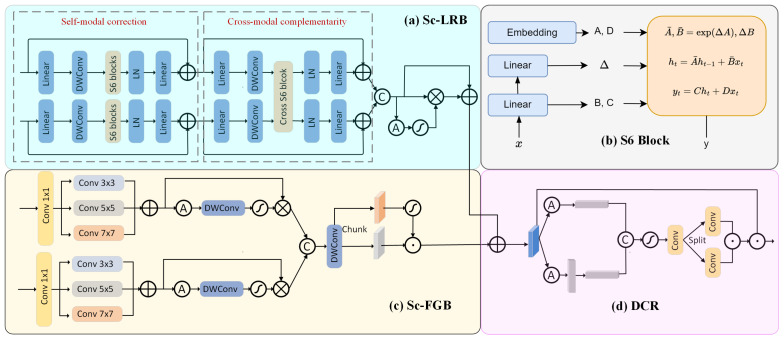
The overall framework of Sc-CFM, consisting of (**a**) a Self-correction Long-Range Relationship Branch (Sc-LRB), (**b**) the principle of the S6 block, (**c**) a Self-correction Fine-Grained Branch (Sc-FGB), and (**d**) Dual-branch Collborative Recalibration (DCR).

After passing through the weight-shared backbone, the RGB image and infrared image will each generate multimodal feature maps $F^{R}=\left\{F_{1}^{R},\ldots ,F_{n}^{R}\right\}$ and $F^{I}=\left\{F_{1}^{I},\ldots ,F_{n}^{I}\right\}$. To effectively couple the above features, we propose the Self-correction and Collaboration Fusion Module (Sc-CFM), as shown in Figure 3. The Sc-CFM refines visual and semantic representations across modalities and harmonizes them for joint optimization. This results in a fused feature map containing rich visual effects and segmentation information. The module comprises three components: Self-correction Long-Range Relationship Branch (Sc-LRB), Self-correction Fine-Grained Branch (Sc-FGB), and Dual-branch Collaborative Recalibration (DCR) for recalibrating the information from different branches.

##### Sc-LRB

We propose a Self-correction Long-Range Relationship Branch (Sc-LRB) based on Mamba to address the efficiency issue in establishing long-range relationships in segmentation tasks. Although ViT has demonstrated outstanding performance in various visual tasks [37], enhancing contextual understanding for semantic segmentation, its self-attention mechanisms are computationally intensive. To address this issue, we introduce the efficient S6 structure [12] and construct our self-correction module for long-range relationship modeling. Consequently, the cross-attention mechanism [38] can capture interrelationships between positions in the input, thereby greatly facilitating feature extraction and complementarity between different data sources. Therefore, our Sc-LRB incorporates two correction strategies: self-modal correction and cross-modal complementarity.

In the self-modal correction stage, the Sc-LRB first receives visible and infrared feature maps from the lower-level encoder and processes them separately through a linear layer and depthwise separable convolution for initial transformation. Subsequently, each of these two feature streams is fed into the S6 module, where linear projection layers are used to generate corresponding matrices (such as *B*, *C*, and Δ) for systematically capturing global dependencies. The module’s output is normalized via layer normalization (LN), passed through a linear projection to restore dimensionality, and finally, added to the original input via residual connection, thereby actively correcting intra-modal feature deviations. The formula can be represented as follows:

(3) $$O_{i}^{r}=F_{i}^{R}+Linear\left(LN\left(S6\left(DWConv\left(Linear\left(F_{i}^{R}\right)\right)\right)\right)\right),$$

(4) $$O_{i}^{i}=F_{i}^{I}+Linear\left(LN\left(S6\left(DWConv\left(Linear\left(F_{i}^{I}\right)\right)\right)\right)\right),$$

Then, we obtain the self-corrected feature map $O^{R}=\left\{O_{1}^{r},\ldots ,O_{n}^{r}\right\}$ and $O^{I}=\left\{O_{1}^{i},\ldots ,O_{n}^{i}\right\}$. For cross-modal complementarity, it takes the corrected dual-modality features as input, with most processing steps similar to those in the previous stage. The difference lies in our custom-designed Cross S6 block, which is capable of simultaneously processing both streams of information, thereby enabling cross-modal information complementation at this stage. The process can be formulated as follows:

(5) $$W_{i}^{r}=O_{i}^{r}+Linear\left(LN\left(crossS6\left(DWConv\left(Linear\left(O_{i}^{r},O_{i}^{i}\right)\right)\right)\right)\right)$$

(6) $$W_{i}^{i}=O_{i}^{i}+Linear\left(LN\left(crossS6\left(DWConv\left(Linear\left(O_{i}^{r},O_{i}^{i}\right)\right)\right)\right)\right)$$

The obtained completed features can be described as: $W^{R}=\left\{W_{1}^{r},\ldots ,W_{n}^{r}\right\}$ and $W^{I}=\left\{W_{1}^{i},\ldots ,W_{n}^{i}\right\}$. The specific architecture of the module is shown in Figure 3a, and Figure 3b illustrates the working principle of the S6 module. For the Cross S6 block, inspired by [39], we implement the corresponding functionality by replacing the matrix C between different modalities, with the following formula:

(7) $$\left.\left\{\begin{array}{cc} {\bar{A}}_{1},{\bar{B}}_{1} & =\exp\left(\Delta A_{1}\right),\Delta B_{1},\\ {\bar{A}}_{2},{\bar{B}}_{2} & =\exp\left(\Delta A_{2}\right),\Delta B_{2}, \end{array}\right.\right.$$

(8) $$\left\{\begin{array}{cc} h_{1}^{t} & =\bar{A_{1}}h_{1}^{t-1}+\bar{B_{1}}x_{1}^{t},\\ h_{2}^{t} & =\bar{A_{2}}h_{2}^{t-1}+\bar{B_{2}}x_{2}^{t}, \end{array}\right.$$

(9) $$\left\{\begin{array}{c} y_{1}^{t}=C_{2}h_{1}^{t}+D_{1}x_{1}^{t},\\ y_{2}^{t}=C_{1}h_{2}^{t}+D_{2}x_{2}^{t}, \end{array}\right.$$

where $A_{1/2}$ and $B_{1/2}$ are the state transition and input matrices, and $C_{1/2}$ and $D_{1/2}$ are the output projection matrices and the direct input-to-output projection matrices for each modality. $x_{1/2}^{t}$ represents the input at time step *t*, and $y_{1/2}$ is the selective scan output. In the SSM, the matrix C is used to map the current state to the output space, determining how the state generates the model’s output. By exchanging the matrix C between different data, we achieve cross-enhancement of features. Subsequently, we obtain the initial fused features $F^{s}=\left\{F_{1}^{s},\ldots ,F_{n}^{s}\right\}$ by concatenating $W^{R}$ and $W^{I}$ and use a channel-wise attention mechanism to extract key information from the fused features, while suppressing low-density information, which can be formulated as follows:

(10) $$\alpha_{i,j}=\frac{1}{H_{i}W_{i}}\sum_{h=1}^{H_{i}}\sum_{w=1}^{W_{i}}F_{i}^{s}\left(h,w,j\right),$$

(11) $$F_{i}^{S}=F_{i}^{s}⊙\sigma \left(\underset{1\times 1}{\mathrm{Conv}}\left(\alpha_{i}\right)\right)+F_{i}^{s},$$

where $\alpha_{i}=\left[\alpha_{i,1},\ldots ,\alpha_{i,C_{i}}\right]\in R^{1\times 1\times C_{i}}$ is the average pooling results of each feature map in $F_{i}^{s}$. Finally, we obtain the fused segmentation images $F^{S}=\left\{F_{1}^{S},\ldots ,F_{n}^{S}\right\}$ after long-range relationship correction.

##### Sc-FGB

In addition to semantic features, fine-grained and rich visual features also play a crucial role in the accurate analysis of details such as edges and steps. Compared to Transformers, convolutional neural networks (CNNs) are more efficient at mining local features and can significantly capture fine-grained details of images. Therefore, we have designed a multi-kernel convolution-based Self-correction Fine-Grained Branch (Sc-FGB) to enhance the extraction of fine-grained features, with specific details shown in Figure 3c. Similar to the Sc-LRB, it receives multimodal feature maps from the shared encoder and outputs a corrected and fused map enriched with abundant visual information and fine-grained features. Specifically, inspired by SegNext [40], we first apply multi-scale convolutions (3×3,5×5,7×7) to perform lateral correction of feature maps from different modalities and then sum these feature maps, which can be formulated as follows:

(12) $$\left\{\begin{array}{cc} H_{i}^{R/I(3)}= & {\mathrm{Conv}}_{3\times 3}\left({\mathrm{Conv}}_{1\times 1}\left(F_{i}^{R/I}\right)\right),\\ H_{i}^{R/I(5)}= & {\mathrm{Conv}}_{5\times 5}\left({\mathrm{Conv}}_{1\times 1}\left(F_{i}^{R/I}\right)\right),\\ H_{i}^{R/I(7)}= & {\mathrm{Conv}}_{7\times 7}\left({\mathrm{Conv}}_{1\times 1}\left(F_{i}^{R/I}\right)\right), \end{array}\right.$$

(13) $${\tilde{H_{i}}}^{R/I}=H_{i}^{R/I(3)}+H_{i}^{R/I(5)}+H_{i}^{R/I(7)},$$

where ${\tilde{H_{i}}}^{R}$ and ${\tilde{H_{i}}}^{I}$ represent the combined characteristics of visible and infrared modalities, respectively. Next, we introduce a channel attention weighting mechanism to integrate information from different scales and obtain an initial visual fusion map $F^{v}=\left\{F_{1}^{v},\ldots ,F_{n}^{v}\right\}$ through concatenation. The formulas are as follows:

(14) $$\begin{array}{cc} A_{i}= & \sigma \left(\mathrm{DWConv}(\mathrm{GAP}\left({\tilde{H}}_{i}^{R/I}\right))\right),\\ {\hat{H}}_{i}^{R/I}= & A_{i}\;⊗\;{\tilde{H}}_{i}^{R/I},\\ F_{i}^{v}= & \mathrm{Concat}({\tilde{H}}_{i}^{R},{\tilde{H}}_{i}^{I}), \end{array}$$

For the concatenated result, we further apply depthwise separable convolutions to correct the details and expand the number of channels. Then, we split it along the channel dimension into two parts, $F^{\alpha }$ and $F^{\beta }$, to capture detailed features from multiple perspectives. Finally, these two parts interact through Hadamard multiplication, achieving the final feature fusion. This process can be represented as follows:

(15) $$F_{i}^{V}=\underset{1\times 1}{\mathrm{Conv}}\left(F^{\alpha }⊙\sigma \left(F^{\beta }\right)\right),$$

Then, we obtain the fused visual feature $F^{V}=\left\{F_{1}^{V},\ldots ,F_{n}^{V}\right\}$ after fine-grained correction.

##### DCR

To synergistically recalibrate the semantically corrected and visually corrected feature streams produced by the preceding branches, we introduce the Dual-branch Collaborative Recalibration (DCR) module (shown in Figure 3d). The two corrected feature sets, $F^{S}$ and $F^{V}$, are first merged via element-wise summation to form a base feature map. To incorporate spatial structural cues, a coordinate attention-inspired mechanism is employed [41]: global average pooling is performed separately along the height and width axes, yielding (H,1) and (1,W) descriptors. Each descriptor is then projected through a 1×1 convolution into an intermediate channel space and passed through a nonlinear activation to produce spatial attention maps. These maps are broadcast across the fused feature map and multiplied elementwise to amplify structurally salient regions and suppress noise. The resulting recalibrated feature representation retains both semantic and visual coherence and serves as the final output of the fusion process. The formulas can be represented as follows:

(16) $$F_{i}^{*}=F_{i}^{S}+F_{i}^{V},\;F^{*}=\left\{F_{1}^{*},\ldots ,F_{n}^{*}\right\},$$

(17) $$\alpha_{i,j,p}^{h}=\frac{1}{W}\sum_{0\leq w<W}F_{i}^{*}\left(p,w,j\right),$$

(18) $$\beta_{i,j,q}^{w}=\frac{1}{H}\sum_{0\leq h<H}F_{i}^{*}\left(h,q,j\right),$$

where $F_{i}^{*}$ is the sum result between $F_{i}^{S}$ and $F_{i}^{V}$, $\alpha_{i}^{h}\in R^{H\times 1\times C_{i}}$ and $\beta_{i}^{w}\in R^{1\times W\times C_{i}}$ store the average pooling results of each feature map. Then, through feature map concatenation and convolution operations, we obtain a feature map that contains rich spatial information, which can be written as follows:

(19) $$W_{i}=\sigma \left(\underset{1\times 1}{\mathrm{Conv}}\left(\left[\alpha_{i}^{h},\beta_{i}^{w}\right]\right)\right),$$

Next, through a split operation, we separately send the horizontal and vertical feature maps into independent convolutional layers, and use activation functions to generate spatial attention weights. Then, these attention weights are applied to the original input feature map, thereby enhancing the spatial dimension of the feature map, which can be represented as follows:

(20) $$F_{i}=F_{i}^{*}⊙{\hat{\alpha }}_{i}^{h}⊙{\hat{\beta }}_{i}^{w},$$

where ${\hat{\alpha }}_{i}^{h}$ and ${\hat{\beta }}_{i}^{w}$ are the split results of $W_{i}$. Finally, the fused images after collaborative recalibration can be defined as: $F=\{F_{1},\ldots ,F_{n}\}$.

#### 3.2.2. ICRM

**Figure 4:**
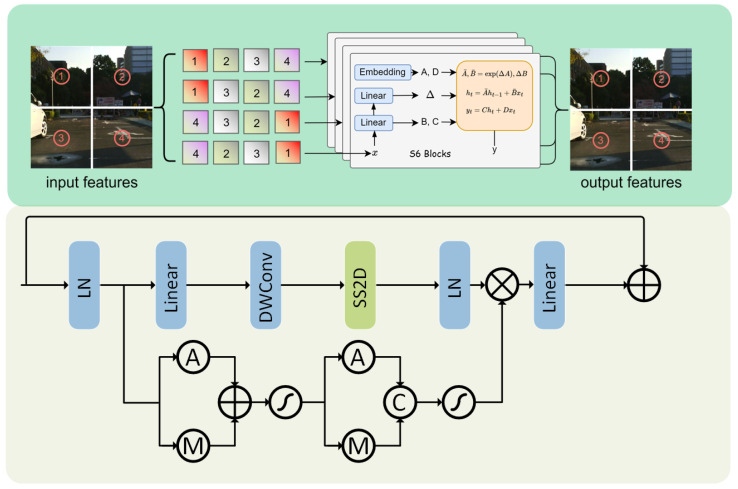
The Interactive Context Restoration Visual State Space Block (ICRVSSB) used in the Mamba decoder. The lower part of the image shows the architecture of ICRVSSB, while the upper part presents the structure of SS2D.

We employ an Interactive Context Restoration Mamba Decoder (ICRM) to decode the feature maps output by Sc-CFM, in order to generate the final prediction result. To fully extract rich local context from the encoder and to mitigate global-context loss during upsampling, we introduce the Mamba architecture as our decoder. The decoder progressively strengthens the relationships between different features in the image through an interactive information restoration process. In this process, each feature map is first subjected to an upsampling operation and then passed through multiple Interactive Context Restoration Visual State Space Blocks (ICRVSSBs) for feature extraction and upsampling; the architecture is shown in the lower part of Figure 4. Finally, the encoded features are passed into the MLP layer for classification, resulting in semantic segmentation output. Our ICRVSSB module is an improvement upon the VSSB architecture based on Vmamba [15], which can be formulated as follows:

(21) $$\Upsilon_{1}=\mathrm{LN}\left(SS2D\left(\mathrm{SiLU}\left(\mathrm{DWConv}\left(\mathrm{Linear}(\Upsilon )\right)\right)\right)\right),$$

where Υ is the feature map fed into the VSSB. The core mechanism of VSSB is the Selective Scan 2D (SS2D) module, as shown in Figure 4 upper part. In SS2D, the 2D data are first unfolded into sequences along four different traversal paths (top-left to bottom-right, bottom-right to top-left, top-right to bottom-left, and bottom-left to top-right). Then, each sequence is processed in parallel through an independent S6 module. The processed result sequences are reshaped and merged to ultimately obtain the output map. Through this process, SS2D is able to integrate pixel information from different directions within the image, effectively enhancing and restoring the global context in the 2D space.

While existing VSSBs excel at extracting global context, they still fall short in capturing channel and spatial information. Therefore, we replace the multiplicative branch in the original VSSB with a channel and spatial attention mechanism. The formula is shown as follows:

(22) $$\Upsilon_{2}=\sigma \left(\Theta^{C}\left(\Theta^{S}\left(\Upsilon \right)\right)\right),$$

where $\Theta^{C}$ represents channel attention and $\Theta^{S}$ represents spatial attention. Finally, we fuse the outputs of the two branches through Hadamard product interaction and use a linear layer to map the channels back to the size of the input channels, which can be written as follows:

(23) $$\Omega =\mathrm{Linear}(\Upsilon_{1}⊙\Upsilon_{2}).$$

The above operations enhance the model’s information interaction and context restoration capabilities, thereby constructing a more robust decoder module.

#### 3.2.3. ReAW

In order to suppress irrelevant information and address feature redundancy, we propose the Region-adaptive Weighted Reconstruction Decoder (ReAW), which further processes the feature maps output by Sc-CFM to produce the final fused image. This allows for the dynamic extraction of region-specific features from the input feature maps. Meanwhile, we believe that the original input image contains important information for image reconstruction, which can guide the more abstractly extracted feature maps. During the feature selection and exclusion process, this guidance helps the feature maps make more reasonable decisions, ultimately achieving better fused image reconstruction. Therefore, in the final image fusion, we incorporate the relevant information from the original image into the final results.

For feature maps with rich visual effects, we first dynamically assign the number of regions to focus on for each feature map. Then, the incoming feature maps are processed by convolution operations of different scales, denoted as e(·), for feature extraction based on the number of regions. The function e(·) can be represented by the following formula:

(24) $$e\left(F_{i}\right)=\mathrm{RELU}\left(\mathrm{BN}\left(\mathrm{Conv}\left(F_{i}\right)\right)\right),$$

Then, two linear layers and a ReLU activation function are used to compute the weight coefficients for different convolutions, thereby dynamically determining which regions need to be focused on. A Softmax function is applied to normalize the coefficients. After obtaining the normalized coefficients, the extracted features corresponding to each region are multiplied by their respective feature weights, and the weighted features of all regions are summed to obtain the total weighted fusion feature, which can be formulated as follows:

(25) $$b_{j}\left(F_{i}\right)=\lambda_{j}e_{j}\left(F_{i}\right),$$

(26) $$B_{i}=\left[b_{1},b_{2},\ldots ,b_{l}\right],$$

where $\lambda_{j}$ is the weight of j-th region, *l* is the number of regions, and $B_{i}$ represents the result after processing the feature map $F_{i}$. Subsequently, each feature $B_{i}$ is upsampled to the original image size for further processing. Finally, the multi-scale weighted feature fusion result is concatenated with the original image features and then fed into a reconstruction layer, which consists of two convolutional layers and activation functions, to obtain the final image fusion result.

### 3.3. Loss Function

In this chapter, we mainly discuss the configuration of the loss functions used during task training, including the segmentation loss for the semantic segmentation task and the fusion loss for image fusion.

**Segmentation loss:** The conventional cross-entropy loss is used to supervise semantic segmentation, which can be formulated as follows:

(27) $$L=-\frac{1}{H\times W}\sum_{h=1}^{H}\sum_{w=1}^{W}\sum_{c=1}^{C}y_{h,w,c}\log\left({\hat{p}}_{h,w,c}\right),$$

Here, *C* is the number of classes, $y_{h,w,c}$ is the one-hot encoding of the true label for the pixel (h,w) and class *c*, and ${\hat{p}}_{h,w,c}$ is the predicted probability by the model that the pixel (h,w) belongs to class *c*.

**Fusion loss:** To generate images with rich visual effects, we design the image fusion loss following [42]. Specifically, we first generate positive samples of the input images through common data augmentation strategies (such as gamma transformation and contrast stretching). Then, we construct the fusion loss from multiple aspects by combining content loss, gradient loss, and color loss. In terms of content loss, the L1 loss between the fused image and the larger value between the corresponding infrared and visible light image positive samples is calculated to ensure that the fusion image retains the most significant parts of both input images. The formula is as follows:

(28) $$L_{\mathrm{con}}=\frac{1}{N}\sum_{i=1}^{N}\left|F_{i}-Max\left(J_{i}^{R},J_{i}^{I}\right)\right|,$$

where *N* is the number of pixels, $J_{i}^{R/I}$ is the pixel value of the RGB image and infrared image, and $F_{i}$ is the pixel value of the fused image.

For the gradient loss, we compute the L1 distance between the gradients of the fused image and the maximum gradients of the corresponding augmented visible and infrared images in both horizontal and vertical directions. Specifically,

(29) $$L_{\mathrm{grad}}=\frac{1}{N}\sum_{i=1}^{N}\left(|\nabla F_{i}-\nabla J_{i}{|}_{x}+{\left|\nabla F_{i}-\nabla J_{i}\right|}_{y}\right),$$

where $\nabla F_{i}$ and $\nabla J_{i}$ represent the gradient of fusion image and the source image.

Finally, the color loss is calculated by the L1 loss between the color channels (Cr and Cb channels) of the fused image and the positive sample in the YCbCr color space, which can be written as follows:

(30) $$L_{\mathrm{color}}=\frac{1}{N}\sum_{i=1}^{N}\left(|C_{r,F_{i}}-C_{r,J_{i}}\left|+\right|C_{b,F_{i}}-C_{b,J_{i}}|\right),$$

where $C_{r,F_{i}}$ and $C_{b,F_{i}}$, respectively, represent the $C_{r}$ and $C_{b}$ channel values of the fused image at pixel i; $C_{r,J_{i}}$ and $C_{b,J_{i}}$ represent the $C_{r}$ and $C_{b}$ channel values of the source image at pixel i. The overall fusion loss is achieved through the weighted sum of the three losses mentioned above. The formula is as follows:

(31) $$L_{\mathrm{total}}=\lambda_{con}L_{\mathrm{con}}+\lambda_{grad}L_{\mathrm{grad}}+\lambda_{color}L_{\mathrm{color}}.$$

## 4. Experiment

### 4.1. Datasets and Implementation Details

#### 4.1.1. Datasets

The proposed method was validated on the MFNet [43] and FMB [17] datasets, which contain 1569 and 1500 pairs of visible and infrared images, respectively, with 393 and 280 pairs used for the test sets. The MFNet dataset includes semantic annotations for 9 classes, while the FMB dataset provides semantic annotations for 14 classes.

#### 4.1.2. Implementation Details

All experiments are conducted on a single NVIDIA 3090 GPU. We jointly trained both tasks for 500 epochs. We employ the AdamW optimizer with an initial learning rate 6 × 10^−5^. In addition, some common data augmentation strategies (random flipping, random scaling, etc.) were also applied in our training. Additionally, infrared information is replicated across the channel dimension three times during data preprocessing to match the H×W×3 input shape of RGB images. The shared encoders are configured as CNN-based structures following ConvNeXt [44], with the number of blocks set to 4 based on its original configuration.

### 4.2. Results of Semantic Segmentation

**Figure 5:**
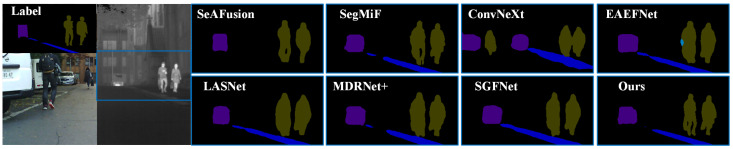
Qualitative segmentation on the MFNet dataset.

**Figure 6:**
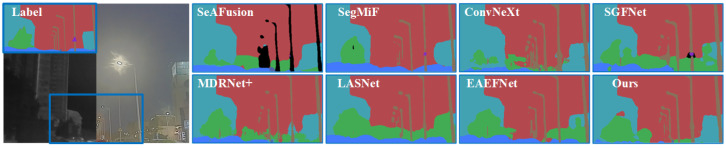
Qualitative segmentation on the FMB dataset.

We provide comparative experiments for semantic segmentation, evaluating against seven state-of-the-art competitors: ConvNeXt [44], SeAFusion [16], SegMiF [17], SGFNet [45], LASNet [46], EAEFNet [47], and MDRNet+ [48]. The qualitative results are depicted in Figure 5 and Figure 6. The experimental results clearly demonstrate the significant advantages of our proposed method in semantic segmentation tasks. For example, as shown in Figure 5, our method preserves pedestrian contours more clearly, demonstrating a more accurate semantic decision-making capability, while the compared methods only achieve vague or approximate region segmentation. Among all the compared methods, except for SeAFusion and SegMiF, none are able to detect the background between persons. Moreover, in terms of contour segmentation accuracy, our method significantly outperforms existing approaches. Furthermore, in Figure 6, our method exhibits superior segmentation performance in terms of fine details (such as lamps). Specifically, compared with existing methods, our approach demonstrates significant advantages in the detection and segmentation of lamp objects. It consistently preserves salient features and achieves accurate detection in long-range scenarios, where other methods often suffer from missed detections or insufficient recognition. Moreover, in close-range scenes, our method captures fine contour details and achieves more precise segmentation, while the compared methods yield approximate contours with limited detail. These results clearly illustrate the superiority of our approach in multi-scale object preservation and fine-grained segmentation.

We also conducted a quantitative comparison using the mean Intersection over Union (mIoU) as the performance evaluation metric. To align with previous experiments, we selected eight categories from the FMB dataset as our evaluation targets. As shown in Table 1 and Table 2, the model we proposed achieved the highest mIoU scores on all test datasets. Overall, our method achieves an improvement of 0.9% in mIoU over the current state-of-the-art methods on the MFNet dataset, and a 1.4% gain in accuracy on the FMB dataset, further validating its effectiveness and generalizability. Specifically, our model achieved the highest IoU values in the Bump category in Table 1 and the Pole category in Table 2, indicating that our model has a significant advantage in segmenting small objects. These results fully demonstrate that our method has reached the current state-of-the-art level in semantic segmentation tasks.

### 4.3. Results of Image Fusion

**Figure 7:**
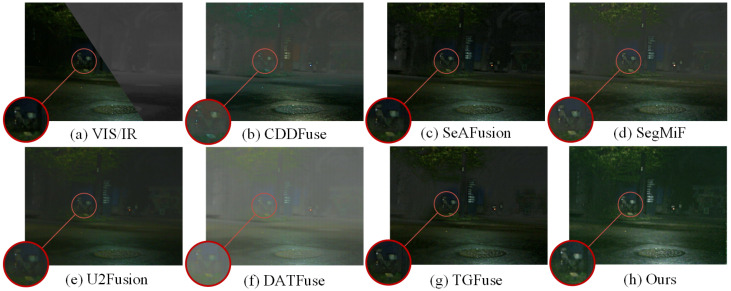
Qualitative comparison results on the FMB dataset.

**Figure 8:**
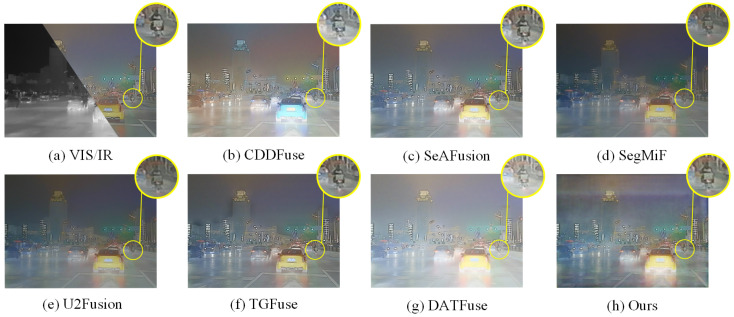
Qualitative comparison results on the FMB dataset.

The performance of image fusion is evaluated through comparative experiments involving six advanced fusion methods, namely U2Fusion [49], SeAFusion [16], SegMiF [17], CDDFuse [10], TGFuse [50], and DATFuse [51]. The qualitative comparison results are shown in Figure 7 and Figure 8, from which we can clearly see that our method demonstrates significant advantages. In Figure 7, our approach significantly improves environmental contrast and object visibility (e.g., leaves and distant buildings) in nighttime image fusion, and it more effectively highlights the detailed features of shadowed objects. In contrast, other methods often lose their ability to perceive the environment under conditions of low visibility. In addition, Figure 8 demonstrates another advantage of our method: Guided by semantic information, our approach can significantly enhance the contrast between the detected objects and their surrounding environment, thereby effectively improving the texture details in the scene and presenting a clearer and more realistic visual effect. In contrast, other methods fail to highlight the boundaries and details of objects as prominently when processing similar scenes, which affects the overall image quality and visualization.

**Figure 9:**
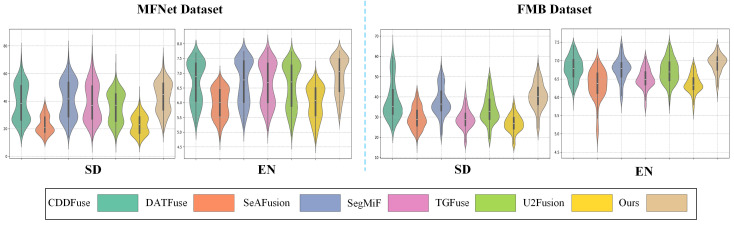
Quantitative analysis results of image fusion.

Due to the lack of ground-truth data, we adopted no-reference metrics EN [52] and SD [53] to evaluate the amount of information and contrast. As shown in Figure 9, SC-CoSF achieved the highest scores on most metrics. In addition, the widest section of the violin plot is concentrated in the upper-middle part of the curve, further confirming that our method consistently achieves superior fusion performance across the majority of images. The above results demonstrate the advanced performance of our method in image fusion.

### 4.4. Complexity Analysis

To evaluate the computational complexity of various semantic segmentation and image fusion methods, we report the number of parameters and FLOPs based on input images with a resolution of 600 × 800 (see Table 3). Although SC-CoSF does not achieve the lowest values in these metrics, its complexity remains within a practically acceptable range and is lower than the complexity of several task-specific segmentation or fusion models. This analysis underscores the practicality of SC-CoSF and provides valuable insights for balancing accuracy and efficiency, especially in scenarios with strict real-time requirements such as autonomous driving and robotic perception systems.

### 4.5. Ablation Study

**Figure 10:**
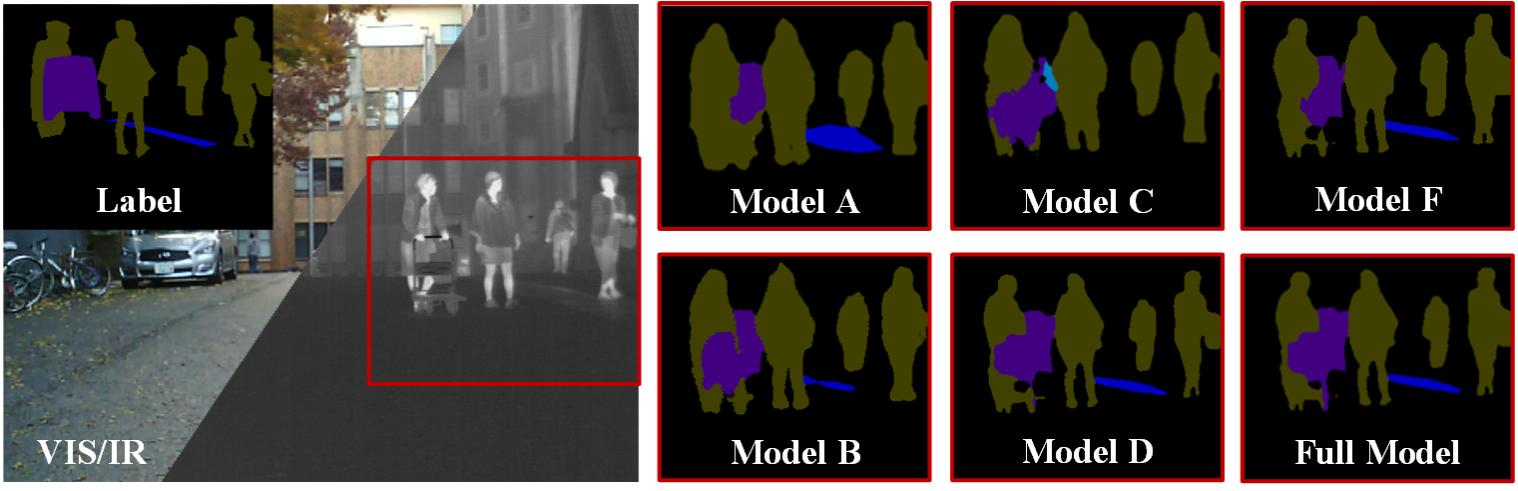
Qualitative segmentation of ablation studies.

**Figure 11:**
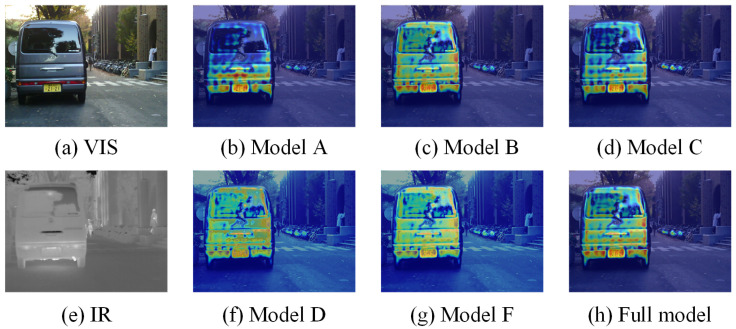
Attention heatmap visualizations of ablation studies.

We conducted extensive ablation experiments on the MFNet dataset to verify the effectiveness of our proposed correction module. In addition, we designed corresponding ablation experiments for different task heads to validate the effectiveness of joint learning. Specifically, our model includes the following variants: **Model A:** Replacing Sc-CFM with tensor addition; **Model B:** Removing Sc-FGB; **Model C:** Removing Sc-LRB; **Model D:** Replacing DCR with tensor addition; **Model E:** Removing the semantic segmentation head; **Model F:** Removing the image fusion head. It is worth noting that for variants E and F, we only conducted the corresponding ablation experiments for the tasks they are associated with. The qualitative and quantitative results of semantic segmentation are shown in Table 4 and Figure 10. It can be seen that removing any module affects the model’s performance. Notably, the results of variants A and C show a significant decrease in contour accuracy for objects (such as pedestrians), highlighting the importance of Sc-LRB in the semantic segmentation task, as it provides strong global contextual capabilities. Additionally, we introduce attention heatmap visualizations in Figure 11 to intuitively compare how each module influences the segmentation process. The results clearly illustrate that the attention heatmaps of models A and C fail to encompass the complete outline of the vehicle, further underscoring the superior performance of Sc-LRB in semantic segmentation tasks.

**Figure 12:**
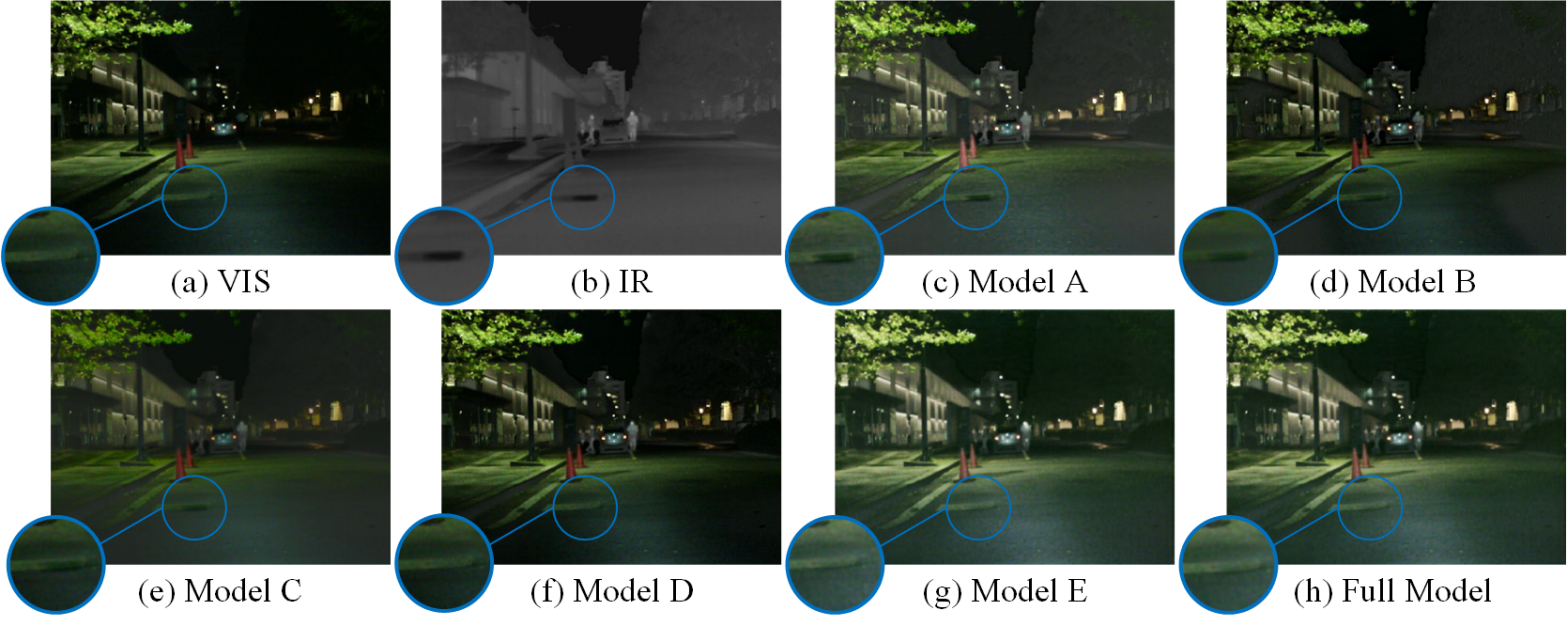
Qualitative fusion of ablation studies.

**Figure 13:**
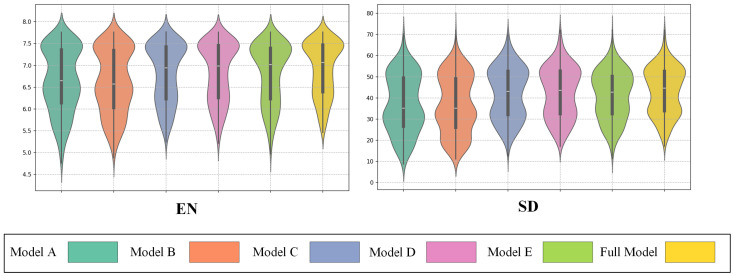
Quantitative fusion of ablation studies.

Figure 12 and Figure 13 present the performance of different variants in image fusion, showing that each module plays a crucial role in the final results. For example, replacing either Sc-LRB or Sc-FGB would make it difficult for the model to recognize and handle the shadows at crossings. These results demonstrate the positive impact of our design on both semantic segmentation and image fusion.

## 5. Discussion

In this study, we propose the SC-CoSF architecture to address multimodal image fusion and semantic segmentation challenges, showing significant advantages for autonomous driving and robotics. Our experiments indicate that by employing a shared-weight backbone and the Self-correction and Collaboration Fusion Module (Sc-CFM), the model effectively integrates heterogeneous information, improving both fusion quality and segmentation accuracy. Unlike conventional methods that treat multimodal inputs independently, our approach leverages the Interactive Context Restoration Mamba Decoder (ICRM) and Region Adaptive Weighted Reconstruction Decoder (ReAW) to exploit the inherent relationships between visual and semantic cues. This end-to-end, multi-task learning mechanism optimizes individual tasks and enhances the overall robustness in dynamic environments.

Despite these promising outcomes, SC-CoSF has limitations that require further investigation. First, its generalization to rare or occluded scenarios needs validation on larger, more diverse datasets. Second, the depth and complexity of our two-branch architecture add computational overhead, potentially hindering real-time performance on embedded platforms. Finally, while the shared-weight strategy reduces parameter count, more lightweight or adaptive fusion schemes could be explored. Future work will (1) evaluate SC-CoSF on real-world driving and robotics benchmarks, (2) investigate model compression and hardware-aware optimization for faster inference, and (3) extend the framework to incorporate temporal coherence for video-based fusion and segmentation.

In summary, the SC-CoSF architecture offers a novel solution for multimodal image fusion and semantic segmentation, providing both theoretical insights and practical benefits. We anticipate that further improvements will advance the state-of-the-art in this rapidly evolving field.

## 6. Conclusions

In this work, we investigated the interdependence between multimodal image fusion and semantic segmentation in autonomous driving and robotics. To address performance limitations, we present SC-CoSF, an innovative framework that enables synergistic learning between these tasks. Our approach introduces three key contributions: First, we develop the Self-correction and Collaboration Fusion Module (Sc-CFM), which includes Sc-LRB for global context modeling, Sc-FGB for local detail preservation, and DCR for cross-modal feature calibration. Second, we design a dual-decoder system with ICRM for long-range dependency recovery and ReAW for adaptive feature reconstruction. Third, we employ an end-to-end joint optimization strategy, using shared parameters to synchronize gradient propagation across tasks, fully exploiting the complementarity between image fusion and semantic segmentation. Quantitative evaluations on multiple public datasets show that SC-CoSF outperforms traditional approaches that optimize tasks independently, improving both fused image quality and segmentation accuracy. Additionally, the collaborative training mechanism enhances inter-modal consistency, increasing model stability and robustness across diverse scenarios. These results demonstrate the potential of tightly coupled multimodal learning in advancing perception capabilities for autonomous driving and robotics.

## Figures and Tables

**Table 1:** Quantitative segmentation on the MFNet dataset.

MFNet	Car	Person	Bike	Curve	Car Stop	Guar.	Cone	Bump	mIoU
SeAFusion	84.2	71.1	58.7	33.1	20.1	0.0	40.4	33.9	48.8
LASNet	84.2	67.1	56.9	41.1	39.6	18.9	48.9	40.1	54.9
SegMiF	87.8	71.4	63.2	47.5	31.1	0.0	48.9	50.5	56.1
MDRNet+	87.1	69.8	60.9	47.8	37.8	6.2	57.1	56.0	56.8
SGFNet	88.4	77.6	64.3	45.8	31.0	0.6	57.1	55.0	57.6
ConvNeXt	89.1	71.9	62.3	44.3	43.0	0.0	51.7	52.6	57.0
EAEFNet	87.6	72.6	63.8	48.6	35.0	14.2	52.4	58.3	58.9
**Ours**	**90.7**	**74.1**	**65.9**	**47.1**	**45.7**	**1.8**	**55.6**	**59.2**	**59.8**

**Table 2:** Quantitative segmentation on the FMB dataset.

FMB	Car	Person	Truck	T-Lamp	T-Sign	Buil.	Vege.	Pole	mIoU
SeAFusion	76.2	59.6	15.1	34.4	68.0	80.1	83.5	38.4	51.9
MDRNet+	79.6	61.3	20.7	19.4	71.5	82.2	85.5	44.0	55.9
SegMiF	79.0	31.1	25.9	49.1	74.7	80.1	84.6	49.5	56.0
LASNet	79.4	54.7	32.2	23.2	70.6	82.1	86.1	45.3	56.1
ConvNeXt	75.0	63.0	36.0	30.1	65.3	82.4	83.2	44.8	57.4
EAEFNet	83.3	65.4	30.6	23.8	72.5	83.9	86.3	48.6	59.7
SGFNet	78.1	68.9	45.0	45.0	72.1	83.1	85.8	45.4	60.4
**Ours**	**77.5**	**67.4**	**39.3**	**46.7**	**72.3**	**83.4**	**85.0**	**52.9**	**61.8**

**Table 3:** Complexity and parameters of segmentation and fusion methods.

Method	FLOPs (G)	Params (M)	Task
MDRNet+	891.82	210.87	Segmentation
LASNet	371.03	93.58	Segmentation
ConvNeXt	278.49	114.36	Segmentation
EAEFNet	316.49	147.21	Segmentation
SGFNet	225.63	125.12	Segmentation
CDDFuse	863.22	1.19	Image Fusion
DATFuse	8.68	0.01	Image Fusion
TGFuse	137.34	19.34	Image Fusion
U2Fusion	633.09	0.66	Image Fusion
SeAFusion	102.53	13.06	Segmentation & Image Fusion
SegMiF	526.20	45.60	Segmentation & Image Fusion
**Ours**	**304.52**	**139.62**	**Segmentation & Image Fusion**

**Table 4:** Quantitative segmentation of ablation studies.

Model	Car	Person	Bike	Curve	Car Stop	Guar.	Cone	Bump	mIoU
A	88.0	71.4	65.5	42.2	40.3	0.2	50.4	48.1	56.0
B	90.0	73.7	64.3	45.5	40.0	0.7	52.3	62.4	58.6
C	88.8	61.0	64.6	39.1	42.4	1.7	51.9	58.5	56.2
D	90.5	73.7	66.1	46.0	39.1	5.3	55.3	61.0	59.5
F	90.5	73.8	66.0	43.8	40.6	1.1	54.8	63.9	59.2
**Full Model**	**90.7**	**74.1**	**65.9**	**47.1**	**45.7**	**1.8**	**55.6**	**59.2**	**59.8**

## Data Availability

Data are contained within the article.
